# Assessment of Serum Macrophage Migration Inhibitory Factor (MIF) as an Early Diagnostic Marker of Leptospirosis

**DOI:** 10.3389/fcimb.2021.781476

**Published:** 2022-02-14

**Authors:** Krishnamoorthi Sumaiya, Charles Solomon Akino Mercy, Gangatharan Muralitharan, Abdurahman Hajinur Hirad, Abdullah A. Alarfaj, Kalimuthusamy Natarajaseenivasan

**Affiliations:** ^1^ Medical Microbiology Laboratory, Department of Microbiology, Centre for Excellence in Life Sciences, Bharathidasan University, Tiruchirappalli, India; ^2^ Department of Botany & Microbiology, College of Science, King Saud University, Riyadh, Saudi Arabia; ^3^ Department of Neural Sciences, Lewis Katz School of Medicine, Temple University, Philadelphia, PA, United States

**Keywords:** leptospirosis, macrophage migration inhibitory factor, diagnostic marker, MIF ELISA, lipopolysaccharide

## Abstract

The search for valuable early diagnostic markers for leptospirosis is ongoing. The aim of the present study was to evaluate the diagnostic value of macrophage migration inhibitory factor (MIF) for leptospirosis. MIF is an immunoregulatory cytokine secreted by a variety of cell types involved in immune response and the pathogenesis of various diseases. It was previously described as a severity predictor of diseases. Samples of 142 leptospirosis cases, 101 other febrile cases, and 57 healthy controls were studied. The prevalence of leptospirosis was 47.3%. Autumnalis, Australis, and Canicola were the highly prevalent leptospiral serovars with a microscopic agglutination test (MAT) titer in the range 1:80–1:2,560. Enzyme-linked immunosorbent assay (ELISA) of MIF was carried out to measure the serum MIF levels. We found that the serum MIF levels [median, (interquartile range)] were significantly (*p* < 0.001) elevated in different clinical forms of leptospirosis, such as febrile illness [7.5 ng/ml (5.32–8.97)], pulmonary hemorrhage [13.2 ng/ml (11.77–16.72)], Weil’s syndrome [8.8 ng/ml (7.25–9.95)], and renal failure [8.6 ng/ml (7.18–10.5)], than in healthy controls [0.65n g/ml (0.5–1.1)]. Serum MIF had sensitivity, specificity, positive predictive value, and negative predictive value of 100%, >90%, >90%, and 100%, respectively. Receiver operating characteristic (ROC) analysis revealed that the serum MIF levels between leptospirosis cases and control subjects had an area under the curve (AUC) value of >0.9 (*p* < 0.0001). In leptospirosis patients, elevation of serum MIF was significantly (*p* < 0.001) higher in severe cases with organ dysfunction [10 ng/ml (7.8–14.5)] than that in mild febrile cases [7.5 ng/ml (5.32–8.97)], with the difference of 2.5 indicating that serum MIF acts as a predictor of leptospirosis severity. Pearson’s correlation test demonstrated that the serum MIF level was strongly correlated (*r* = 0.75, *p* < 0.0001) with disease progression. The median lethal dose (LD_50_) of leptospiral lipopolysaccharide (LPS) in BALB/c mice was determined to be 20 mg/kg, which gave rise to endotoxemia. Leptospiral LPS triggered the upregulation of MIF expression at 24 h post-infection, which reached the peak level at 24 h post-treatment in THP-1 cells and showed elevated MIF expressions in different tissues of BALB/c mice at the early stage of infection. Taken together, MIF is an early-phase cytokine that could serve as a rapid diagnostic marker for leptospirosis.

## Introduction

Leptospirosis is a spirochaetal zoonotic disease caused by pathogenic leptospires infecting both humans and animals. It has a spectrum of clinical presentations ranging from self-limiting mild, nonspecific flu-like illness, to severe fatal conditions (Haake and Levett, 2015). The mortality and morbidity remain significant, and every year, 1.03 million people are affected worldwide (Costa et al., 2015). The signs and symptoms of leptospirosis simulate those of well-known diseases such as typhoid, dengue, malaria, and acute hepatitis (Safa et al., 2017), leading to underdiagnosis and inadequate timely treatment (Izurieta et al., 2008). The two major limitations that complicate leptospirosis management are the lack of knowledge on the importance of disease considerations and the lack of early and accurate diagnostic tools. Hence, frequent estimation of the disease prevalence in tropical regions and the development of early diagnostic markers are urgently needed.

Currently, leptospirosis represents a major challenge in the healthcare system. It has been reported throughout the world, especially in tropical and subtropical regions. In India, leptospirosis is endemic in seven states, including Tamil Nadu, Kerala, Karnataka, Maharashtra, and Gujarat, and in one union territory, Andaman and Nicobar Islands, especially in North and South Andaman (Loganathan and Shivakumar, 2008). Natural catastrophes such as flooding and cyclones can increase human exposure to leptospires, which leads to its outbreak (Kobayashi, 2005). During the post-monsoon, the outbreak of leptospirosis occurs almost every year in Andaman Islands, especially in regions where people engage in agricultural activities (Sehgal et al., 1995; Vijayachari et al., 2004; Sugunan et al., 2009). In Kerala, more than 1,000 leptospirosis cases are reported every year, and it has the greatest mortality rate compared to other contagious diseases. Indeed, a noticeable outbreak in Kerala has been recently reported in 2018 (James et al., 2018). Epidemics of leptospirosis are often reported in urban areas, such as Chennai and Mumbai (Bharadwaj et al., 2002; Loganathan and Shivakumar, 2008; Loganathan et al., 2012).

Although it has been reported for decades, the problem of leptospirosis is not well documented in developing countries. Frequent estimation of the disease prevalence and the incidence rates in high-risk regions is required, serving as an important public health tool to prevent and control the disease outbreak (Ward, 2013). As leptospirosis is still underdiagnosed and underreported in Tamil Nadu, our research team has frequently investigated and reported the prevalence of leptospirosis in Tiruchirappalli (Vedhagiri et al., 2013; Prabhakaran et al., 2014; Vanithamani et al., 2015; Raja et al., 2016; Kanagavel et al., 2017), where 70% of the population engage in agriculture and allied activities. The preliminary part of the present study was designed to evaluate the disease burden in Tiruchirappalli from June 2017 to February 2018.

Another crucial complication with leptospirosis is the limitation of a rapid diagnosis (Rajapakse et al., 2015). The standard serological assays are dependent on circulating antibodies and thereby fail to diagnose the early phase of leptospirosis (Toyokawa et al., 2011). Misdiagnosis in the early phase of the disease would lead to the life-threatening severe form of leptospirosis with multi-organ involvement, including renal failure, liver dysfunction, pulmonary hemorrhage, and meningitis, which bring about dramatically increased mortality rates (Cagliero et al., 2018). Previous investigations suggested that the development of rapid diagnostic markers and the initiation of antibiotic therapy can achieve successful treatment of leptospirosis (Toyokawa et al., 2011). Recent research on leptospirosis has mainly focused on the identification of novel biomarkers with high predictive value. Previous studies suggested that host immunological factors can serve as diagnostic markers and are deemed to play critical roles in the progression into severe leptospirosis (Zuerner, 2015). Several host mediators have been investigated as potential biomarkers of leptospirosis, such as human serum (mannose binding lectin, MBL) (Miranda et al., 2009), interleukin 6 (IL-6), IL8, IL-10, soluble suppression of tumorigenicity 2 receptor (Wagenaar et al., 2009a), pentraxin 3, and copeptin (Wagenaar et al., 2009b). In this study, we propose the consideration of serum macrophage migration inhibitory factor (MIF) for the diagnosis of leptospirosis because the expression of MIF is significantly elevated at the early stage of disease induction.

MIF is a pro-inflammatory cytokine that acts as a potential regulator of host response to infection (Calandra and Roger, 2003). MIF is an approx. 12.5-kDa highly conserved secreted protein (Yang et al., 2017) with pro-inflammatory and immunomodulatory activities. It has been involved in several immunological processes such as leukocyte recruitment, inflammation, immune response, cell proliferation, tumorigenesis, and counter-regulation of glucocorticoids (Chen et al., 2017). Growing evidence supports the correlation between serum MIF concentration and the pathogenesis of several inflammatory diseases (Kithcart et al., 2010). More recently, emphasis has been on the use of serum MIF as an early diagnostic marker for diseases to achieve early diagnosis and improve disease management. Previous investigations showed that elevated cytokine production plays a predominant role in the development of severe leptospirosis (Cagliero et al., 2018). In this case–control study, we determined the disease prevalence and proposed the use of serum MIF as a potential early diagnostic tool to successfully combat the outbreak of leptospirosis in developing countries.

## Materials and Methods

### Study Design and Study Site

A case–control study was conducted to estimate the prevalence of leptospirosis in Tiruchirappalli, to determine the serum levels of MIF in leptospirosis patients compared with other febrile cases and healthy control subjects, and to assess the leptospiral lipopolysaccharide (LPS)-mediated MIF upregulation in *in vitro* and *in vivo* experimental models. This study was carried out from June 2017 to February 2018 by active hospital-based surveillance at the Annal Gandhi Memorial General Hospital, Tiruchirappalli, Tamil Nadu, India. The temperature in the study area ranges from 36°C to 41°C, and its geographical position is 10°48′18″ N latitude and 78°41′08″ E longitude.

### Study Population and Case Definition

A total of 300 study subjects were recruited to participate in the study. In total, 243 blood samples from clinically suspected cases with clinical manifestations such as fever, myalgia, body ache, arthritis, icterus, rigors, breathlessness, abdominal pain, conjunctival suffusion, subconjunctival hemorrhages, and jaundice with acute renal failure were collected for the diagnosis of leptospirosis. The samples were collected before any treatment was given to the patients. A total of 57 seronegative healthy controls recruited from the general population in the same geographical area matched for age ( ± 5 years) and sex were included as controls. Healthy control subjects who had fever in the previous 2 weeks were excluded from the study. The obtained serum samples were divided into aliquots and stored at −80°C until the assay was performed.

### Live Antigens and Microscopic Agglutination Test

For serological evidence of leptospirosis in the study population, microscopic agglutination test (MAT) was performed using a panel of 12 live leptospiral serovars. Leptospiral cultures were maintained by regular sub-culturing in Ellinghausen–McCullough–Johnson–Harris (EMJH) medium supplemented with bovine serum albumin and Tween-80 at the Medical Microbiology Laboratory, Bharathidasan University, Tiruchirappalli. The following serogroups were used as live antigens: Australis (serovar Australis, strain Ballico), Autumnalis (serovar Autumnalis, strain Akiyami A), Ballum (serovar Ballum, strain Mus 127), Bataviae (serovar Bataviae, strain Swart), Canicola (serovar Canicola, strain Hond Utrecht IV), Icterohaemorrhagiae (serovar Icterohaemorrhagiae, strain RGA), Grippotyphosa (serovar Grippotyphosa, strain Moskva V), Hebdomadis (serovar Hebdomadis, strain Hebdomadis), Javanica (serovar Poi, strain Poi), Pomona (serovar Pomona, strain Pomona), Pyrogenes (serovar Pyrogenes, strain Salinem), and Sejroe (serovar Hardjo, strain Hardjoprajitno). Seven-day-old live leptospiral culture of 1 × 10^8^ organisms/ml was used as the antigen. Double dilution of serum was performed serially starting from 1:20 and incubated with live antigens for agglutination. A titer of ≥1:160 and agglutination of ≥50% were considered as positive for MAT. Phosphate-buffered saline (PBS) was used as a diluent in the assay.

### IgM Enzyme-Linked Immunosorbent Assay

Immunoglobulin M (IgM) enzyme-linked immunosorbent assay (ELISA) was performed to further confirm leptospiral infection in suspected cases by assessing the early immune response of cases to leptospirosis. Heat-extracted leptospiral antigens were prepared as described earlier (Kanagavel et al., 2017). Of the leptospiral antigens, 0.2 µg was coated on 96-well microtiter plates at appropriate wells using carbonate coating buffer (pH 9.6) and then stored at 4°C for 12 h. Each well was washed three times with PBST (PBS + 0.1% Tween-20) for 10 min each. About 3% blocking solution (non-fat milk) was added to each well and incubated at 37°C for 1 h. Each well was washed, as previously mentioned. The test sera were added into appropriate wells at a dilution of 1:100 and incubated at 37°C for 1 h. After washing the wells, the bound IgM antibody was detected by adding peroxide-conjugated anti-human IgM antibody (1:1,000) and incubated at 37°C for 1 h, followed by developing with *o*-phenylenediamine dihydrochloride (OPD). Fifty microliters of 1 N H_2_SO_4_ was added to stop the reaction, and then the optical density was measured at 490 nm using a microtiter plate reader.

### MIF Immunoassay

Quantitative measurement of human MIF in patient sera was carried out using Human MIF ELISA Kit (Sigma-Aldrich, St. Louis, Mo, USA). All procedures were in accordance with the manufacturer’s instructions. In brief, all reagents and samples were allowed to reach 18–25°C before use. All samples and standards were performed in triplicate. Of the samples, 100 μl was added into microtiter wells and incubated for 3 h at room temperature with gentle shaking. Then, the solution was discarded and the wells washed four times with 1× wash buffer. Subsequently, 100 μl of 1× biotinylated detection antibody was added to each well and incubated for 1 h at room temperature with gentle shaking. Following washing, 100 μl of horseradish peroxidase (HRP)–streptavidin solution was added to each well and incubated for 45 min at room temperature with gentle shaking. The wells were again washed and 100 μl of ELISA colorimetric 3,3′,5,5′-tetramethylbenzidine (TMB) reagent was added to each well and incubated for 30 min at room temperature in the dark. Fifty microliters of the stop reagent was added to each well and the plates immediately read at 450 nm. The mean absorbance values for each set of standards, controls, and samples were calculated and the average zero standard optical densities were subtracted for background correction. The standard curve was plotted using SigmaPlot 11.0 software, with the standard concentration on the *x*-axis and absorbance on the *y*-axis to quantify the MIF. Serum MIF levels were expressed as nanograms per milliliter.

### Mouse-Adapted Challenge Strain

Mouse-adapted challenge strains (MACS) were prepared from their corresponding parent strains (reference laboratory strains/isolates) by passaging them in immunocompromised BALB/c mice (300 mg/kg body weight of cyclophosphamide treatment) ~15 times (Adler and Faine, 1976; Adler and Faine, 1977). The cultures used in all the experiment in the current study were *Leptospira interrogans* serovar Autumnalis strain N2 (human isolate) MACS passaged *in vitro* less than three times (Kanagavel et al., 2017).

### Extraction of LPS From MACS

*L. interrogans* serovar Autumnalis strain N2 MACS were grown in liquid EMJH medium at 30°C and collected at a density of ~5 × 10^8^ leptospires/ml. Leptospiral LPS was extracted with the standard hot phenol–water method (Westphal and Jann, 1965). The phenol phase was purified by dialysis. The extracted LPS were quantified using the phenol/sulfuric acid method (Vanithamani et al., 2015).

### Cell Culture and LPS Induction *In Vitro*

THP-1 cells were purchased from the National Centre for Cell Science, Pune, and cultured in RPMI 1640 medium with 10% fetal bovine serum at 37°C and 5% CO_2_. The cells were pelleted and washed three times with serum-free culture medium. Approximately 5 × 10^5^ cells per well were added into six-well plates and then treated with LPS (1 μg/ml) at different time intervals (0, 5, 10, 20, and 30min and 1, 2, 3, 6, 12, 24, 48, and 60 h). All treatments were performed in triplicate. Treated cells were pelleted for further analysis.

### BALB/c Mice and Determination of LPS LD_50_

An inbred strain of BALB/c mice (weighing about 20 ± 2 g) was used throughout the study. To accustom the mice, they were housed under ambient room temperature (25 ± 2°C), with a 12-h light/dark cycle, and given standard feed and water *ad libitum* over a period of 10 days before the start of the experiments. Mice 4–6 weeks old were separated into five groups. Five mice (immunocompromised) in each group were challenged (intraperitoneally) with different doses (5, 10, 20, and 30 mg/kg) of *L. interrogans* serovar Autumnalis strain N2 for the determination of the survivability of the BALB/c mouse model. Mice injected with PBS were considered as untreated control. Survival over 4 days was frequently evaluated and the LD_50_ of leptospiral LPS determined.

### Detection of Endotoxemia in the Mouse Model

Dot blot assay and cytokine profiling were performed to examine endotoxemia in LD_50_ LPS-injected mice. Blood sample was collected from the control (PBS-injected) and LPS-injected mice and the plasma separated for analysis. Two miroliters of plasma samples and 5 µg purified LPS were loaded into a 0.2-µm nitrocellulose membrane, probed with patient sera (1:100), and incubated for 1 h at room temperature. Anti-human IgM (ALP-conjugated) was added to the membrane and incubated for 1 h. The membrane was washed for 30 min with PBST after every incubation step, developed with the SuperSignal West Pico Chemiluminescent Substrate (Thermo Fisher Scientific, Waltham, MA, USA), and documented in Fusion Solo™ Personal Blot and Gel Imaging System (Vilber Lourmat, Paris, France). Dot intensity was measured densitometrically and expressed in arbitrary units.

Quantitative real-time PCR (qRT-PCR) was carried out to measure the expressions of the inflammatory cytokines [tumor necrosis factor alpha (TNF-α), IL-1β, IL-4, and IL-10] of mice injected with LD_50_ LPS. Monolayer cells from the plasma were collected and RNA was isolated with the RNeasy Mini Kit according to the manufacturer’s instructions (Qiagen, Valencia, CA, USA). Complementary DNA (cDNA) was synthesized and qRT-PCR was performed using a CFX96 Touch™ Real-Time PCR Detection System (Bio-Rad, Hercules, CA, USA). The primers used in this study are listed in **Supplementary Table S1**.

### LPS Induction *In Vivo*

The mice to be injected with leptospiral LPS were primarily immunocompromised with cyclophosphamide (300 mg/kg body weight). The mice were subjected to LPS exposure by intraperitoneal injection. The untreated control group was maintained by injecting with PBS alone. The dosage of LPS was 20 mg/kg body weight. Infected mice were monitored every 12 h for clinical outcomes and survival up to 8 days. Dead and moribund animals were euthanized for harvesting the tissues. Vital organs including the heart, lungs, kidney, spleen, and liver and lymphoid organs such as the thymus, bone marrow, spleen, and lymph node were collected and stored in liquid nitrogen for MIF profiling experiments.

### Protein Extraction and Western Blotting

The collected THP-1 cells and homogenized tissues were washed three times with ice-cold PBS. Cell lysates were prepared using ice-cold RIPA lysis buffer (50 mM Tris–HCl, pH 7.4, 150 mM NaCl, 0.25% deoxycholic acid, 1 mM EDTA, and 1% NP-40) (Thermo Fisher Scientific, Waltham, MA, USA) and stored at −20°C. The extracted protein was quantified using the bicinchoninic acid (BCA) method. Thirty micrograms of protein per well was loaded onto 12% polyacrylamide gel and electrophoresed at 60 V in Mini-PROTEAN Tetra System (Bio-Rad, Hercules, CA, USA). The separated proteins were transferred into a nitrocellulose membrane (pore size, 0.2 µm) electrophoretically at 12 V for 1 h using a V20 semi-dry blotter (Scie-Plas, Cambridge, UK). Subsequently, the membranes were blocked with a blotting grade blocking solution (5%, *w*/*v*) at room temperature for 1 h, washed three times for 10 min each with 1× TBST (TBS + 0.1% Tween-20), and incubated with rabbit anti-MIF antibody (1:1,000; Invitrogen, Carlsbad, CA, USA) at 4°C for 12 h. Bound antibodies were detected by incubating with HRP-conjugated anti-rabbit antibody (1:5,000; Sigma-Aldrich, St. Louis, MO, USA). Bands were developed with the West Pico Signal Chemiluminescence developing kit (Thermo Fisher Scientific, Waltham, MA, USA) and documented in Fusion Solo™ Personal Blot and Gel Imaging System (Vilber Lourmat, Paris, France). Band intensities were calculated using ImageJ software, and the data were normalized to the loading control.

### Quantitative Real-Time PCR Analysis

RNA was extracted from the harvested tissues and THP-1 cells using an RNeasy Mini Kit according to the manufacturer’s instructions (Qiagen, Valencia, CA, USA). The concentration and the purity of mRNA were determined using the BioPhotometer Plus system (Eppendorf, Hamburg, Germany). cDNA was synthesized using the iScript cDNA synthesis kit (Bio-Rad, Hercules, CA, USA). qRT-PCR was performed using a CFX96 Touch™ Real-Time PCR Detection System (Bio-Rad, Hercules, CA, USA) The primers used in this study are listed in **Supplementary Table S1**. The qRT-PCR using SYBR Green PCR Master Mix (Bio-Rad, Hercules, CA, USA) and primers was carried out in a 10-μl reaction volume (20 ng cDNA, 5 μl Master Mix, and 0.2 μM of each primer). GAPDH was used as the loading control.

### Statistical Analysis

Data from triplicate experiments were quantified and expressed as the mean ± SD (*n* = 3). Serum MIF levels with outlier points were expressed as median (interquartile range, IQR). Data were computed either with GraphPad Prism version 9.2.0 or SigmaPlot 11.0 software. Receiver operating characteristic (ROC) analyses were performed to estimate the sensitivity and specificity of MIF as a diagnostic marker for leptospirosis. Pearson’s correlation coefficient test was performed to assess the correlation of serum MIF with disease duration and patient age. A two-tailed paired Student’s *t*-test or the Mann–Whitney *U* test was performed to analyze differences between the study groups. Kaplan–Meier plots were generated using GraphPad Prism version 7.0 to quantify survivability. A *p*-value ≤0.05 was considered significant.

### Ethics Statement

The studies involving human participants (for the collection of blood samples) were reviewed and approved by the Institutional Ethical Committee (no. DM/2014/101/51) of Bharathidasan University. Informed consent was obtained from both patients and healthy controls prior to the sample collection; in the case of minor study participants, their surrogates signed an informed consent form. The animal experimental protocols were approved by the Institutional Animal Ethical Committee (BDU/IAEC/P30/2018), Bharathidasan University.

## Results

### Seroprevalence of Leptospirosis

The seroprevalence of leptospirosis was determined with the MAT assay with the panel of 12 leptospiral serovars as an antigen. Out of the 300 serum samples tested for antibodies against pathogenic *Leptospira*, a total of 142 cases tested positive at agglutination titer of ≥1:80, giving an overall seroprevalence of 47.3%. Seropositivity was uniformly distributed in both genders and in all age groups. The demographic characteristics of the clinical subjects included in the study are shown in **Table 1**.

**Table 1:** Demographic characteristics of the study subjects.

S. no.	Variables		Confirmed leptospirosis cases	Other febrile cases	Heathy control subjects
1	Age (years)	Range	6–75	5–75	8–74
		Mean ± SD	34.2 ± 17.7	28 ± 16.8	35.8 ± 18.0
2	Sex (%)	Female	48	43.5	54.4
		Male	52	56.5	45.6
3	Duration of disease (days)	Range	7–31	2–41	–
		Mean ± SD	19.8 ± 7	11.9 ± 7.5	–
4	Serology	MAT titers (range)	1:80–1:2,560	–	–
		IgM ELISA titer	1:100	–	–

MAT, microscopic agglutination test; IgM, immunoglobulin M.

Of the 12 leptospiral serovars tested, six were detected among the samples from the study subjects. The prevalent infecting serovars were Autumnalis (50.7%), followed by Australis (21.2%), Canicola (16.2%), Icterohaemorrhagiae (15.6%), Grippotyphosa (3.5%), and Ballum (2.9%). MAT titers were reported to be in the range between 1:80 and 1:2,560. The MAT-positive titers with the respective serovars are represented in **Table 2**. Among the 142 patients who tested positive for MAT, 9 (6.3%) cases were negative for IgM ELISA.

**Table 2:** Serovar distribution and MAT titers in the study subjects.

Serovar	Frequency, *n* (%)	Median MAT titers	1:80 (%)	1:160 (%)	1:320 (%)	1:640 (%)	1:1280 (%)	1:2560 (%)
Autumnalis	72 (50.7)	1:640	12.5	13.8	16.6	33.3	16.6	6.9
Australis	30 (21.1)	1:640	16.6	16.6	13.3	26.6	23.3	3.3
Canicola	23 (16.2)	1:320	17.3	17.3	26	26	8.6	4.3
Icterohaemorrhagiae	8 (15.6)	1:640	12.5	12.5	0	50	12.5	12.5
Grippotyphosa	5 (3.5)	1:320	20	20	40	20	20	0
Ballum	4 (2.9)	1:160	25	25	50	0	0	0

MAT, microscopic agglutination test.

### Elevated Serum MIF in Leptospirosis Cases

**Figure 1:**
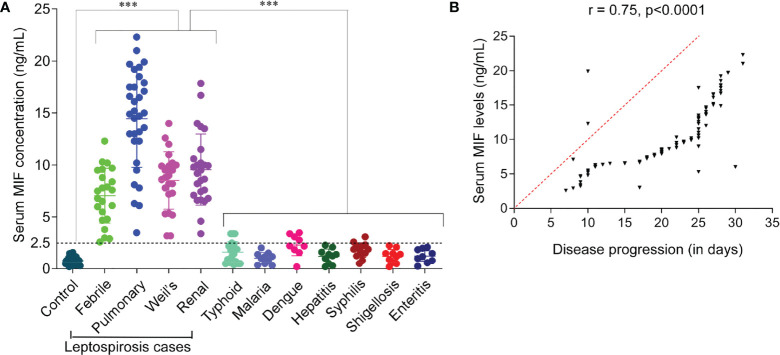
Serum macrophage migration inhibitory factor (MIF) profiling and analysis of its diagnostic value for the early diagnosis of leptospirosis. **(A)** Profiling of MIF in sera of patients with leptospirosis with different clinical manifestations, other febrile illnesses, and healthy controls. Study groups are indicated on the *x*-axis and the MIF concentration on the *y*-axis. *Dotted line* represents the cutoff with the absolute values on the *left*. *n* = 3 experiments. ***p < 0.001. **(B)** Pearson’s correlation coefficients between serum MIF levels and disease progression. This analysis showed that serum MIF was positively correlated with disease progression, which indicates the contribution of serum MIF to disease severity (*r* = 0.75, *p* < 0.0001), acting as a severity predictor.

To identify whether MIF protein was differentially expressed between leptospirosis patients and healthy control subjects, we used the sera of the study subjects to assess circulating MIF concentrations with MIF ELISA. The cutoff point for a positive MIF level was 0.008 ng/ml. As shown in **Figure 1A**, it was found that the serum MIF levels were significantly (*p* < 0.001) elevated in different clinical conditions of patients with leptospirosis, including febrile illness (median = 7.5 ng/ml, IQR = 5.32–8.97), pulmonary hemorrhage (median = 13.2 ng/ml, IQR = 11.77–16.72), Weil’s syndrome (median = 8.8 ng/ml, IQR = 7.25–9.95), and renal failure (median = 8.65 ng/ml, IQR = 7.18–10.5), compared to those in healthy controls (median = 0.65 ng/ml, IQR = 0.5–1.1) and other febrile cases such as typhoid (median = 1.32 ng/ml, IQR = 0.57–1.9), malaria (median = 1.1 ng/ml, IQR = 0.57–1.35), dengue (median = 2.2 ng/ml, IQR = 1.58–2.8), hepatitis (median = 1.32 ng/ml, IQR = 0.62–1.56), syphilis (median = 1.9 ng/ml, IQR = 1.46–2.2), shigellosis (median = 1.31 ng/ml, IQR = 0.67–1.5), and enteritis (median = 1.2 ng/ml, IQR = 0.8–1.85). Patients with pulmonary hemorrhage have remarkably higher MIF levels than with other clinical manifestations. The MIF levels of all study subjects are presented in **Table 3**.

**Table 3:** Serum macrophage migration inhibitory factor (MIF) levels in patients and healthy controls.

Study subjects (*n*)	Serum MIF level (ng/ml)		
	Range	Median (interquartile range)	*p*-value
Laboratory-confirmed leptospirosis cases (142)			
Febrile illness (41)	2.6–12.3	7.5 (5.32–8.97)	<0.001
Pulmonary hemorrhage (37)	3.5–22.3	13.2 (11.77–16.72)	<0.001
Weil’s syndrome (35)	3.2–14	8.8 (7.25–9.95)	<0.001
Renal syndrome (29)	3.4–17.85	8.65 (7.18–10.5)	<0.001
Other febrile cases (101)			
Typhoid (40)	0.4–3.4	1.62 (0.57–1.9)	1
Malaria (10)	0.32–2.03	1.1 (0.57–1.35)	1
Dengue (9)	0.2–3.5	2.23 (1.58–2.8)	1
Hepatitis (10)	0.2–2.3	1.32 (0.62–1.56)	1
Syphilis (13)	0.52–3.1	1.9 (1.46–2.2)	1
Shigellosis (10)	0.2–2.25	1.31 (0.67–1.5)	1
Enteritis (9)	0.25–2.1	1.2 (0.8–1.85)	1
Healthy controls (57)	0.2–1.6	0.65 (0.5–1.1)	–

### ROC Curve Analysis of MIF as a Candidate Biomarker

To estimate the diagnostic value of serum MIF as a biomarker for leptospirosis, ROC curve analysis was performed. ROC analysis revealed that the serum MIF levels discriminated significantly between leptospirosis cases and healthy control subjects. The area under the curve (AUC) of the different clinical manifestations of leptospirosis was >0.9 (*p* < 0.0001) (**Supplementary Figures S1A–D**). An AUC value >0.9 was considered as an outstanding quality of discrimination between groups. The optimal cutoff value for MIF was predicted as 2.5 ng/ml by stressing the higher sensitivity. The sensitivity and the specificity of serum MIF profiling for leptospirosis cases were found to be 100% and >90%, respectively, in different clinical forms of leptospirosis. The positive predictive value (PPV) and the negative predictive value (NPV) were >90% and 100%, respectively. The sensitivity/specificity values and the PPV/NPV for the different clinical manifestations of leptospirosis are shown in **Table 4**. Our results demonstrated that the levels of serum MIF were significantly upregulated in leptospirosis patients compared to those in healthy control subjects and that it will be possible to use MIF as a biomarker to improve disease monitoring and management.

**Table 4:** Receiver operating characteristic (ROC) analysis of macrophage migration inhibitory factor (MIF) as an early diagnostic marker for leptospirosis.

Clinical parameters	Sensitivity (%)	Specificity (%)	PPV (%)	NPV (%)	AUC ± SE	*p*-value
Febrile illness	100	91.7	91.54	100	0.9910 ± 0.006	<0.0001
Pulmonary hemorrhage	100	99	98.9	100	0.9999 ± 0.0003	<0.0001
Weil’s syndrome	100	95.4	95.12	100	0.9960 ± 0.003	<0.0001
Renal syndrome	100	95.4	95.12	100	0.9989 ± 0.001	<0.0001

PPV, positive predictive value; NPV, negative predictive value; AUC, area under the curve.

### Serum MIF Level Correlates With Leptospirosis Disease Progression and Severity

The difference in the serum MIF levels between leptospirosis cases with febrile illness (mild, non-hospitalized) and those with organ dysfunction (severe, hospitalized) was significantly high (2.5, *p* < 0.001). Our results revealed that the serum MIF levels were more significantly elevated in patients with severe than in those with mild leptospirosis. Here, we analyzed whether there was any association between serum MIF and leptospirosis disease progression. We evaluated the serum MIF levels associated with disease progression using Pearson’s correlation test and found a significantly high positive correlation (*r* = 0.75, *p* < 0.0001). A correlation coefficient index (*r*) of ≥0.7 was considered as a high positive correlation. There was no significant correlation found in the serum MIF levels of study subjects in respect to age (*r* = 0.006). An *r* value of <0.3 was considered as negligible correlation between variables. The correlation of elevated serum MIF with the disease progression of leptospirosis is represented in **Figure 1B**. The analysis suggested that serum MIF may serve as a severity predictor of human leptospirosis.

### Determination of LPS LD_50_ and Endotoxemia in BALB/c Mice

**Figure 2:**
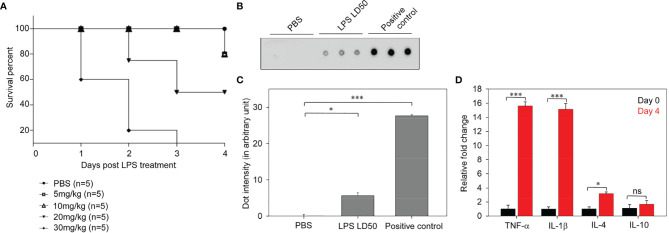
Determination of lipopolysaccharide (LPS) median lethal dose (LD_50_) of *Leptospira interrogans* serovar Autumnalis strain N2 in BALB/c mice. **(A)** Representative Kaplan–Meier plot of LD_50_ determinations showing the survivability at different doses (5, 10, 20, and 30 mg/kg) of leptospiral LPS-injected mice. **(B)** Dot blot assay of the LD_50_ (10 mg/kg) of leptospiral LPS causing endotoxemia in infected mice. Plasma samples of mice injected with PBS and LD_50_ LPS were assayed. Purified LPS was loaded as a positive control. **(C)** Representative graph of the dot intensity of samples. **(D)** Analysis of the expressions of inflammatory cytokines in the leptospiral LPS-injected mouse model by qRT-PCR. *n* = 3 experiments. *p < 0.05; ***p < 0.001, ns, no significant.

For the determination of LD_50_, LPS-challenged mice were frequently evaluated for disease progression and moribund state. The number of deceased mice was recorded to plot the Kaplan–Meier survival graph. The LD_50_ for leptospiral LPS was found to be 20 mg/kg, which caused the death of about 50% of the mice. Mice challenged with 5, 10, and 30 mg/kg of LPS exhibited 80%, 80%, and 20% of survival, respectively (**Figure 2A**). Thus, we used 20 mg/kg of leptospiral LPS for subsequent experiments.

The dot blot immunoassay detected the significant occurrence of LPS in the blood stream of infected mice. This confirmed that the intraperitoneal injection of LD_50_ LPS induced endotoxemia in the mouse model (**Figures 2B, C** **)**. Endotoxemic inflammation after injection of LD_50_ LPS was characterized by analysis of the cytokine expressions. The qRT-PCR results showed a significant (15-fold) increase of the expressions of TNF-α and IL-1β and a threefold increase of the expression of IL-4 (**Figure 2D**). The high expressions of pro-inflammatory cytokines in LPS-infected mice revealed endotoxemic inflammation. Therefore, the administration of leptospiral LD_50_ LPS induced endotoxemic inflammation in BALB/c mice.

### MIF Profiling *In Vitro* and *In Vivo*

**Figure 3:**
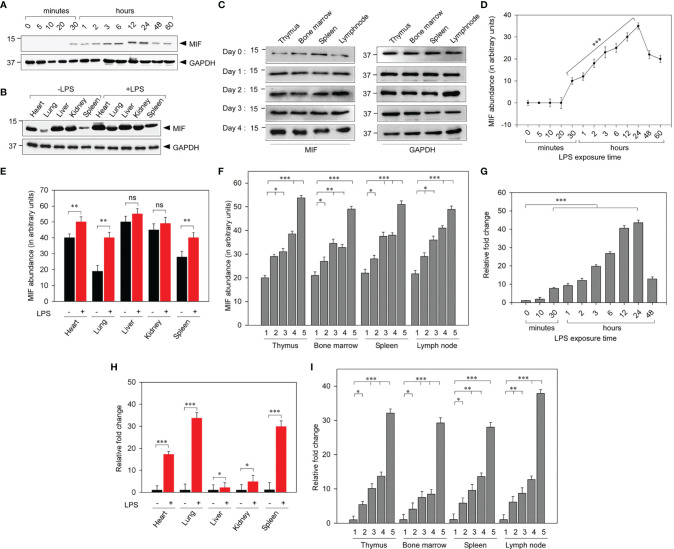
Analysis of the macrophage migration inhibitory factor (MIF) profile in leptospiral lipopolysaccharide (LPS)-induced experimental models. **(A)** Representative Western blot analysis of the increased expression of MIF in LPS-treated THP-1 cells in a time-dependent manner. **(B)** Representative Western blot analysis of the significantly upregulated expression of MIF in the lungs and spleen among the vital organs of LPS-induced BALB/c mice. **(C)** Representative Western blot analysis of the progressive MIF upregulation in primary and secondary lymphoid organs at different time intervals. **(D–F)** Quantification of MIF abundance by densitometric measurement from **(A–C)**, respectively. **(G–I)** Analyses of leptospiral LPS-stimulated MIF mRNA expressions in THP-1 cells **(G)** and in vital organs **(H)** and lymphoid organs **(I)** of BALB/c mice by qRT-PCR. *1*, day 0; *2*, day 1; *3*, day 2; *4*, day 3; *5*, day 4. *n* = 3 experiments. *p < 0.05; **p < 0.01; ***p < 0.001. ns, no significant.

To demonstrate the MIF expression profile during the early phase of leptospiral infection, we have experimentally induced leptospiral infection in *in vitro* and *in vivo* models. Cell lysates prepared from THP-1 cells treated with leptospiral LPS (1 μg/ml) were assayed using Western blotting to determine the intracellular MIF profile (**Figures 3A, D** **)**. MIF expression was at the baseline in untreated cells. Leptospiral LPS significantly (*p* < 0.001) increased the expression level of MIF in a time-dependent manner. The expression of MIF in THP-1 cells was markedly increased at 30 min post-treatment, which gradually upregulated and reached the peak level at 24 h post-treatment and then significantly diminished at 60 h.

To assess the elevated MIF expression in lymphoid organs and other vital organs during leptospiral pathology, tissue samples were harvested from leptospiral LPS-administered BALB/c mice and were assayed with Western blotting. The results showed significantly (*p* < 0.01) upregulated MIF expression in vital organs post-LPS injection, particularly in the lung, spleen, and heart compared to other organs (**Figures 3B, E** **)**. As MIF is an immune mediator, we investigated the regulation of its expression in both primary and secondary lymphoid organs, including the thymus, bone marrow, spleen, and lymph node, on days 0, 1, 2, 3, and 4 post-LPS treatment. The expression of MIF was significantly increased in all lymphoid organs on days 1, 2, 3, and 4 post-LPS treatment when compared with day 0. In untreated control mice, differential MIF expression was observed in vital organs, whereas no significant difference in its expression was noticed between lymphoid organs (**Figures 3C, F** **)**.

Furthermore, RT-PCR analysis confirmed the LPS-mediated upregulated *MIF* gene expression by measuring the increased MIF mRNA levels. Leptospiral LPS gradually upregulated the *MIF* gene expression in a time-dependent manner in LPS-treated THP-1 cells (**Figure 3G**). As *in the vitro* MIF protein profile, the *MIF* gene expression was remarkably increased in the lung, spleen, and heart of LPS-injected mice (**Figure 3H**). *MIF* gene expression analysis on lymphoid organs at different time intervals revealed that the mRNA expression was gradually increased in all lymphoid organs up to day 3 and drastically increased on day 4. No significant morphological changes were observed in the lymphoid organs of mice injected with LD_50_ LPS (**Figure 3I**). These results confirmed that MIF is an early-phase secreted cytokine and that its use as a biomarker will be a promising early diagnostic tool for leptospirosis. Taken together, the mRNA and protein expressions of MIF were significantly increased as the duration of infection increased, with MIF possibly driving the pathogenesis of leptospirosis.

## Discussion

We began this study with the determination of leptospirosis prevalence in Tiruchirappalli district. It was found that evaluation of the serum MIF levels of patients has diagnostic implications for leptospirosis. To the best of our knowledge, our study revealed, for the first time, that MIF-specific ELISA may be a promising biomarker for the early diagnosis of leptospirosis. In this study, we also explored the MIF protein expression profile in leptospiral LPS-induced *in vitro* and *in vivo* models to confirm MIF as an early-phase secreted cytokine. We are aware that our research may have a limitation, which is the period of clinical investigation from 2017 to 2018. Therefore, the seroprevalence of leptospirosis may not match those of current investigations.

Leptospirosis is a neglected tropical zoonotic disease with a high disease burden and high mortality rates in developing countries due to the limited knowledge of physicians on leptospirosis, difficulty of obtaining early diagnosis, and the delayed initiation of effective treatments. The primary step of disease management is to attain knowledge regarding the disease prevalence in the area and develop an early diagnostic tool. Here, we reported the significant percentage of disease burden and the prevalent serovars, which included Autumnalis, Australis, Canicola, Icterohaemorrhagiae, Grippotyphosa, and Ballum. This report addressed the importance of disease consideration in tropical regions and increased the research base of leptospirosis to accomplish complete documentation of the disease. Our study also suggested that the prevalent serovars should be included in the panel of MAT antigens in clinical laboratories of the study area in order to minimize false-negative results.

Currently, various serological tests are available for the diagnosis of leptospirosis, such as MAT, ELISA, macroscopic agglutination test, microcapsule agglutination, and the dipstick assay (Vanithamani et al., 2015). Despite being the gold standard reference for leptospirosis, serogroup-specific MAT assay is complex to perform and has shown technical limitations and low sensitivity in patients with the early phase of the disease. Indeed, MAT exhibited sensitivity values of 41%, 82%, and 96% in the first, second to the fourth, and after the fourth week of the onset of disease, respectively (Musso and La Scola, 2013). During an outbreak, these complex methods are not suitable for evaluating a large quantity of samples. A novel diagnostic biomarker that has high sensitivity and specificity and is affordable is urgently required in regions of developing countries with high leptospirosis prevalence in order to combat frequent disease outbreaks. The available diagnostic assays are dependent on the activation of the adaptive immune response, whereas the IgM antibody is detectable only at 5–7 days post-infection, which leads to delayed antibiotic therapy for patients. Leptospirosis patients begin to worsen if they are not properly treated within 2–3 days (Kobayashi, 2005; Musso and La Scola, 2013). A misdiagnosed and untreated early phase of leptospirosis causes cytokine storm and tissue damage, which lead to the development of severe leptospirosis with multi-organ dysfunction (Cagliero et al., 2018). However, the currently evaluated diagnostic marker (MIF) is an early expressed inflammatory cytokine, which may serve as a promising early diagnostic marker for leptospirosis. MIF ELISA exhibited sensitivity of 100% and specificity of more than 90% for leptospirosis.

Previous studies reported a wide range of cytokines secreted by the host immune system during leptospirosis. Especially, TNF-α and the interleukins IL-1β, IL-2, IL-4, IL-6, IL-8, and IL-10 were elevated in severe leptospirosis cases, whereas TNF-α, IL-6, IL-8, IL-10, interferon-γ, and soluble TNF receptor 1 were elevated in high fatality cases (Senavirathna et al., 2020). The hemolysin of *L. interrogans* acts as a pro-inflammatory stimulator that triggers the production of cytokines by the Toll-like receptor 2- and 4-dependent JNK (c-Jun N-terminal kinase) and NF-κB (nuclear factor kappa-light-chain-enhancer of activated B cell) pathways (Wang et al., 2012). Patients’ immune response to leptospirosis, especially the cytokine production, causes the variations of disease outcomes. As inflammatory cytokines are secreted during the early stages of infection, the implication of these cytokines as diagnostic biomarkers facilitates the detection of the acute phase of the disease.

In recent decades, researchers have focused on the contribution of MIF in inflammatory diseases. MIF is an immunoregulatory/inflammatory cytokine that is differentially expressed between patients and healthy controls. Normally, MIF circulates at a concentration of 2 ng/ml in human blood plasma. During infection, the MIF level is drastically elevated, which makes it possible to function as a biomarker for specific diseases (Grieb et al., 2010). The combined determination of MIF and other biomarkers upgrades the detection of fatal outcomes (Grieb et al., 2010). Therefore, MIF was considered in this study to evaluate its diagnostic prospects. In general, the major problem in leptospirosis is its diagnosis, which is still mostly misleading due to the febrile-related symptoms of typhoid, dengue, malaria, hepatitis, enteritis, and shigellosis (Gasem et al., 2020; Md-Lasim et al., 2021). In this study, our observations clearly stated that MIF ELISA has high sensitivity (100%) and specificity (>90%) for the diagnosis and distinction of leptospirosis cases from other febrile cases. A significantly different cutoff point of the serum MIF level (*p* < 0.001) was detected in leptospirosis patients when compared with other cases, which greatly suggested that MIF is a potential early diagnostic marker for leptospirosis. AUC values of 0.5, 0.7–0.8, 0.8–0.9, and >0.9 were considered as showing non-acceptable, acceptable, excellent, and outstanding discriminating ability to diagnose patients with and without the disease (Mandrekar, 2010). MIF had an AUC value of >0.9 for the different outcomes of leptospirosis. According to the ROC analysis, MIF is strongly suggested as an efficient biomarker for rapid diagnostic purposes.

Pearson’s correlation test demonstrated that the serum MIF levels were strongly correlated (*r* = 0.75) with disease duration, directly indicating that MIF might be deeply involved in the pathology of leptospirosis. However, no significant correlation was found with respect to age, which is consistent with previous studies on autoimmune and inflammatory diseases. The degree of infection, microbial count, and host immune responses showed more potential as factors than age, which may have obscured the impact of age on the levels of serum MIF (Mizue et al., 2000; Sreih et al., 2011). The correlation coefficient index (*r*) values of 0.0–0.3, ≥0.3, ≥0.5, ≥0.7, and 0.9–1.0 referred to negligible, low, moderate, high, and very high positive correlations, respectively (Mukaka, 2012). Therefore, MIF not only showed high diagnostic values but was also associated with disease severity. As there is still no remarkable severity predictor for leptospirosis, it is assumed that our significant range of elevated serum MIF may serve as a potential predictor of severe leptospirosis. As severe leptospirosis with multi-organ dysfunction may be fatal, the early prediction of the disease severity and the development of appropriate treatments are needed. Furthermore, certain cases such as severe leptospirosis patients with acute renal failure have not shown clinical response to most treatments. Previous investigations suggested that convalescent plasma therapy may be very useful for the treatment of these cases due to its fast recovery and ability to reduce the bacterial load (Tse et al., 2002). Thus, a therapeutic strategy applicable for the treatment of both mild and severe leptospirosis cases is also urgently needed.

Existing research works have addressed MIF as being implicated in the pathogenesis of various inflammatory and autoimmune diseases, including sepsis (Bozza et al., 2004), rheumatoid arthritis (Kim et al., 2011), diabetes (Sanchez-Zamora et al., 2010), solid tumors and cancer (Nobre et al., 2017), acute respiratory distress syndrome (Donnelly et al., 1997), hepatic inflammatory diseases (Marin et al., 2017), multiple sclerosis (Benedek et al., 2017), systemic lupus erythematosus (Tu et al., 2019), psoriasis (Bezdek et al., 2018), and dengue (Chuang et al., 2015). Leptospiral LPS is a serovar-specific major immunodominant antigen that initiates the pathogenesis of leptospirosis (Vanithamani et al., 2015). The present study determined the LD_50_ (20 mg/kg) of *L. interrogans* serovar Autumnalis strain N2 for the development of the experimental animal model of leptospirosis to analyze the expression profile of MIF during the disease. Endotoxemia and upregulation of pro-inflammatory cytokines were reported in leptospiral LPS-challenged mice. The intraperitoneally injected LPS can cross the gastrointestinal barrier to enter the blood stream. Circulating LPS then bound to the LPS-binding protein and complexed with the CD14 receptor, which can stimulate the pro-inflammatory cytokines (André et al., 2019). Monocytes and macrophages rapidly secreted the MIF protein after exposure to microbial products, especially LPS (Bernhagen et al., 1993; Calandra and Roger, 2003). LPS and TNF stimulation regulated MIF/CD74 signaling to promote B-cell proliferation and inflammation (Klasen et al., 2018) and regulated the expression of TLR4 in fibroblasts (Xi et al., 2016). The immunopathological mechanism underlying MIF expression and its role are not well understood. MIF is an acute-phase secretory protein that appears in the blood at 2–8 h of infection (Calandra et al., 1994).

Our study is the first to investigate the MIF profile in leptospiral LPS-treated *in vitro* and *in vivo* models. THP-1 cells and mice with experimentally induced leptospirosis showed significantly upregulated MIF expression at the early phase of leptospirosis. MIF profiling in untreated control mice showed differential MIF expression in the heart, lung, liver, kidney, and spleen, among which the kidney and liver showed higher MIF expressions. However, our results for the spleen, which is a secondary lymphoid organ, revealed that it had a low MIF expression in normal mice. MIF expression is regulated by several immune mediators, particularly the pro-inflammatory cytokine TNF-α, which enhances the promoter activity of MIF by activating the nuclear transcription factor NF-κB (Cao et al., 2006). Spleen is a notable organ that showed very low TNF-α expression, but the liver and kidney cells exhibited predominant expressions (Hunt et al., 1992). MIF has a wide tissue distribution; specifically, the organs involved in stress response have high levels of MIF expression (Calandra and Roger, 2003).

Upon infection, the expression of MIF was significantly increased in the spleen, lung, and heart. In general, during infection, matured lymphocytes (one of the predominant sources of MIF expression) migrate into the spleen to fight antigens. Thereby, the spleen showed a significantly higher MIF expression during leptospiral infection in mouse models. Alveolar macrophages and the immune cells in mucosa-associated lymphoid tissue (MALT) could be responsible for the elevated MIF expression during infection. The infiltration of immune cells in tissues may play a key role in the differential MIF abundance in different tissues. Besides, we found that the expression of MIF was dramatically increased in all lymphoid organs in response to the duration of infection. As immune cells are the primary sources of MIF secretion during infection, the formation and activation of lymphocytes, macrophages, and other immune cells in primary and secondary lymphoid organs play a key role in MIF expression (Calandra and Roger, 2003). In the present study, the early expression of MIF and its time-dependent increase upon infection suggested a role of this cytokine in disease pathogenesis and progression. Thus far, only very few studies have investigated the mechanism of the contribution of MIF in the pathology of inflammatory diseases. Further studies are required to better understand its role in different inflammatory diseases and in the development of therapeutic regimens.

## Conclusion

Our data provided the first evidence of differential MIF levels in sera of leptospirosis patients, other febrile cases, and healthy control subjects. According to our results, MIF is an early-phase cytokine biomarker that provides positive results in the early phase as possible and exhibits high sensitivity and specificity. Thereby MIF meets the desired attributes of an ideal biomarker. MIF profiling in leptospirosis patients also suggested that an elevated concentration of serum MIF is a superior indicator for predicting severe leptospirosis. *In vitro* and *in vivo* MIF profiling determined the timeline of early MIF gene expression and its differential expression in different organs during leptospiral infection. Thus, the present findings provide the framework for further studies for understanding the role of MIF in leptospirosis.

## Data Availability Statement

The original contributions presented in the study are included in the article/**Supplementary Material**. Further inquiries can be directed to the corresponding author.

## Ethics Statement

The studies involving human participants were reviewed and approved by the Institutional Ethical Committee (no. DM/2014/101/51) of Bharathidasan University. The patients/participants provided written informed consent to participate in this study. The animal study was reviewed and approved by the Institutional Animal Ethical Committee (BDU/IAEC/P30/2018), Bharathidasan University.

## Author Contributions

KS: Conceptualization, Methodology, Software, Writing-Original draft preparation. CM: Conceptualization, Data curation, Methodology, Writing- Original draft preparation. GM: Data curation, Methodology. AH: Visualization, Investigation, Software, Data curation, Methodology, Reviewing and Funding acquisition. AA: Supervision, Reviewing, Editing, Funding acquisition. KN: Conceptualization, Validation, Funding acquisition, Project administration, Writing, Reviewing and Editing.

## Funding

This study was supported by the Indian Council of Medical Research (Leptos/34/2013-ECD-I; Leptos/33/2013-ECD-I), Government of India, Department of Science and Technology (DST/INSPIRE Fellowship/2017/IF170956, Government of India, and the Researchers Supporting Project (RSP-2021/98) of King Saud University, Riyadh, Saudi Arabia. We also acknowledge the funding support of Rashtriya Uchchatar Shiksha Abhiyan (RUSA) 2.0. The authors extend their appreciation to the Researchers supporting Project number (RSP-2021/98), King Saud University, Riyadh, Saudi Arabia for financial support.

## Conflict of Interest

The authors declare that the research was conducted in the absence of any commercial or financial relationships that could be construed as a potential conflict of interest.

## Publisher’s Note

All claims expressed in this article are solely those of the authors and do not necessarily represent those of their affiliated organizations, or those of the publisher, the editors and the reviewers. Any product that may be evaluated in this article, or claim that may be made by its manufacturer, is not guaranteed or endorsed by the publisher.
