# Supplementary material for: Assessment of Serum Macrophage Migration Inhibitory Factor (MIF) as an Early Diagnostic Marker of Leptospirosis
Source: Front Cell Infect Microbiol. 2022 Feb 14;11:781476. doi: 10.3389/fcimb.2021.781476 (PMC8884337; doi:10.3389/fcimb.2021.781476)

Supplementary Material

**Supplementary Table 1.**

**Table S1: List of primer sequences used for gene expression analysis**

| **Gene** | **Sequence** |
| --- | --- |
| **Human MIF: FP** | 5’-CGC AGA ACC GCT CCT ACA G-3’ |
| **Human MIF: RP** | 5’-GGA GTT GTT CCA GCC CAC AT-3’ |
| **Mouse MIF: FP** | 5’-TGC CCA GAA CCG CAA CTA CAG TAA-3’ |
| **Mouse MIF: RP** | 5’-TCG CTA CCG GTG GAT AAA CAC AGA-3’ |
| **Mouse TNF-α: FP** | 5’-GGA CTA GCC AGG AGG GAG AA-3’ |
| **Mouse TNF-α: RP** | 5’-CGC GGA TCA TGC TTT CTG TG-3’ |
| **Mouse IL-1β: FP** | 5’-AAA CAG ATG AAG TGC TCC TTC CAG G-3’ |
| **Mouse IL-1β: RP** | 5’-TGG AGA ACA CCA CTT GTT GCT CCA-3’ |
| **Mouse IL-4: FP** | 5’-CAA ACG TCC TCA CAG CAA CG-3’ |
| **Mouse IL-4: RP** | 5’-AAG CCC GAA AGA GTC TCT GC-3’ |
| **Mouse IL-10: FP** | 5’-GCC CTT TGC TAT GGT GTC CT-3’ |
| **Mouse IL-10: RP** | 5’-TTT TCA GGG ATG AAG CGG CT-3’ |
| **Human GAPDH: FP** | 5’-AAC GAC CCC TTC ATT GAC-3’ |
| **Human GAPDH: FP** | 5’-TCC ACG ACA TAC TCA GCA C-3’ |
| **Mouse GAPDH: FP** | 5’-CTC CCA CTC TTC CAC CTT CG-3’ |
| **Mouse GAPDH: FP** | 5’-GCC TCT CTT GCT CAG TGT CC-3’ |

**Supplementary Figure 1.**

**Figure S1:** ROC curve of MIF for different clinical manifestations of leptospirosis. **(A)** febrile illness (AUC=0.9910), **(B)** pulmonary hemorrhage (AUC=0.9999), **(C)** Weil’s syndrome (AUC=0.9960), **(D)** Renal failure (AUC=0.9989). AUC values represent the diagnostic value of MIF, >0.9 was considered as outstanding ability of biomarker to diagnose the disease.


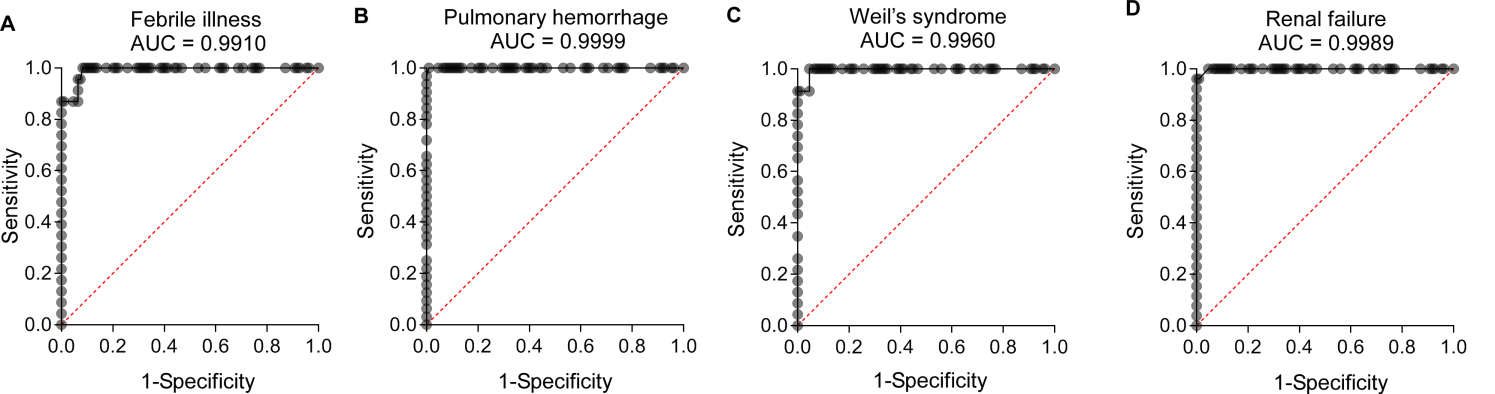

Supplement: Supplementary file 1 [file DataSheet_1.docx]
